# Estimating mother-to-child HIV transmission rates in Cameroon in 2011: a computer simulation approach

**DOI:** 10.1186/s12879-016-1336-2

**Published:** 2016-01-12

**Authors:** Hermine L. Nguena Nguefack, Henri Gwet, Sophie Desmonde, Odile Ouwe Missi Oukem-Boyer, Céline Nkenfou, Mathurin Téjiokem, Patrice Tchendjou, Irénée Domkam, Valériane Leroy, Ahmadou Alioum, for the Inserm U897 Modeling Infectious Diseases in Low-Income Countries Study Group

**Affiliations:** 1 National Advanced School of Engineering, The University of Yaoundé I, PO Box 8390, Yaoundé, Cameroon; 2 Inserm, U897 Bordeaux, France; 3 Bordeaux School of Public Health, The University of Bordeaux, Bordeaux, France; 4 Centre International de Référence Chantal Biya (CIRCB) pour la recherche sur la prévention et la prise en charge du VIH/SIDA, Yaoundé, Cameroun; 5 Centre de Recherche Médicale et Sanitaire (CERMES), member of Réseau International des Instituts Pasteur, Niamey, Niger; 6 Centre Pasteur du Cameroun, member of Réseau International des Instituts Pasteur, Yaoundé, Cameroun; 7 Inserm U1027 Epidémiologie et analyses en santé publique : risques, maladies chroniques et handicaps, Université Paul Sabatier Toulouse 3, Toulouse, France

**Keywords:** HIV, Mother-to-child transmission, Africa, Children, Breastfeeding, Modelling

## Abstract

**Background:**

Despite the progress in the Prevention of the Mother-to-Child Transmission of HIV (PMTCT), the paediatric HIV epidemic remains worrying in Cameroon. HIV prevalence rate for the population of pregnant women was 7.6 % in 2010 in Cameroon. The extent of the paediatric HIV epidemic is needed to inform policymakers. We developed a stochastic simulation model to estimate the number of new paediatric HIV infections through MTCT based on the observed uptake of services during the different steps of the PMTCT cascade in Cameroon in 2011. Different levels of PMTCT uptake was also assessed.

**Methods:**

A discrete events computer simulation-based approach with stochastic structure was proposed to generate a cohort of pregnant women followed-up until 6 weeks post-partum, and optionally until complete breastfeeding cessation in both prevalent and incident lactating HIV-infected women. The different parameters of the simulation model were fixed using data sources available from the 2011 national registry surveys, and from external cohorts in Cameroon. Different PMTCT coverages were simulated to assess their impact on MTCT. Available data show a low coverage of PMTCT services in Cameroon in 2011.

**Results:**

Based on a simulation approach on a population of 995, 533 pregnant women, the overall residual MTCT rate in 2011 was estimated to be 22.1 % (95 % CI: 18.6 %–25.2 %), the 6-week perinatal MTCT rate among prevalent HIV-infected mothers at delivery is estimated at 12.1 % (95 % CI: 8.1 %–15.1 %), with an additional postnatal MTCT rate estimated at 13.3 % (95 % CI: 9.3 %–17.8 %). The MTCT rate among children whose mothers seroconverted during breastfeeding was estimated at 20.8 % (95 % CI: 14.1 %–26.9 %). Overall, we estimated the number of new HIV infections in children in Cameroon to be 10, 403 (95 % CI: 9, 054–13, 345) in 2011. When PMTCT uptake have been fixed at 100 %, 90 % and 80 %, global MTCT rate failed to 0.9 % (95 % CI: 0.5 %–1.7 %), 2.0 % (95 % CI: 0.9 %–3.2 %) and 4.3 % (95 % CI: 2.4 %–6.7 %) respectively.

**Conclusions:**

This model is helpful to provide MTCT estimates to guide the national HIV policy in Cameroon. Increasing supply and uptake of PMTCT services among prevalent HIV infected pregnant women, as well as HIV-prevention interventions including the offer and acceptance of HIV testing and counselling in lactating women could reduce significantly the residual HIV MTCT in Cameroon. A public health effort should be made to encourage health care workers and pregnant women to use PMTCT services until complete breastfeeding cessation.

**Electronic supplementary material:**

The online version of this article (doi:10.1186/s12879-016-1336-2) contains supplementary material, which is available to authorized users.

## Background

It is estimated that 90 % of HIV infections in children are resulting from mother-to-child-transmission (MTCT) [1]. By the end of 2011, of the 34 million adults infected with HIV worldwide, 16 million were women [2]; in sub-Saharan Africa, prevalence of HIV among pregnant women is high. In the absence of any intervention to prevent MTCT (PMTCT), the MTCT rate varies between 13 % and 48 % [1–3]. Maternal combination antiretroviral therapy (ART) together with postnatal interventions have demonstrated their efficacy in reducing substantially the risk of MTCT in African breastfed children to less than 5 % [4, 5]. The World Health Organization (WHO) has called for the “virtual elimination” of pediatric HIV [6, 7] with the most efficient and cost-effective ways to tackle paediatric HIV being to reduce MTCT worldwide [8]. However, access to ART and the uptake of PMTCT programs remain limited and children continue to be HIV-infected [1].

In Cameroon, the prevalence of HIV was estimated to be 4.3 % in the general population; a sero-surveillance survey among pregnant women showed an HIV prevalence of 7.6 % in 2010 [9]. As a result the number of new paediatric infections continues to grow in Cameroon and there are still thousands of new infections each year [10]. In 2011, the UNAIDS launched the Global Plan towards the elimination of new HIV infections among children and keeping their mothers alive [7] making Cameroon, which overall MTCT risk was reported to be around 24 % [7], one of the 21 priority countries. Since 2011, Cameroon has tripled its coverage of PMTCT prophylaxis, ranging from 6.9 % to 36.5 % in 2011, leading to 30 % fewer new HIV infections among children [11]. In 2011, Cameroon opted for the WHO Option A regimen for PMTCT prophylaxis. Continuing access of pregnant women living with HIV to prenatal HIV services and increasing access to HIV treatment for eligible children and pregnant women will reduce maternal and child mortality [11]. Cameroon has focused on strengthening PMTCT services and caring of paediatric HIV cases for the 2011–2015 period: 99.4 % of health districts are equipped to provide HIV treatment services for pregnant women and children living with HIV in 2011. However, even where the most effective PMTCT interventions are available, many women and infants are lost at different steps of the PMTCT cascade [12] and the low cumulative uptake of PMTCT services does not allow controlling the extent of MTCT in Cameroon.

Clinical trials and cohort studies provide essential information in assessing PMTCT interventions; however these studies are also very time and resource-consuming, difficult to implement and not representative. Alternatively, simulation models can integrate available data and help to project long-term outcomes. To date, several published analyses have reported on simulation models to estimate MTCT of HIV. Vieira et al. developed a discrete-event, three-phase simulation, built in Visual Basic, with a stochastic semi-Markov structure to model different intervention strategies at any time, including short-course antiretroviral drugs and cessation of breastfeeding in Botswana [13]. Ciaranello et al. used local trial and programmatic data to simulate a cohort of HIV-infected, pregnant/breastfeeding women in Zimbabwe and to compare five PMTCT regimens at a fixed level of PMTCT medication uptake. The authors linked published computer simulation models to project clinical outcomes of five PMTCT strategies [14]. Another simulation model for MTCT is the Spectrum software package, developed by Stover et al. [15–17]. The Spectrum program is used to forecast key HIV prevalence and PMTCT rates based on national HIV surveillance and surveys, program statistics and epidemic patterns. To the best of our knowledge, to date there are no published models that simulate individually a cohort of pregnant women in Cameroon able to estimate MTCT rates accounting for both prevalent (infected at delivery) and incident (infected in the lactating period) maternal HIV infections. Thus, we developed a stochastic simulation model to estimate the MTCT risk (perinatal and postnatal) and the number of new paediatric infections based on the observed uptake of PMTCT programmes at different stages of the PMTCT cascade in Cameroon in 2011. PMTCT uptake is an important determinant of MTCT, we thus assessed the impact of increase of PMTCT uptake at 100 %, 90 % and 80 % on the MTCT of HIV in Cameroon.

## Methods

We developed a discrete event computer simulation-based approach with a stochastic structure to generate a hypothetical cohort of pregnant women followed-up through different states during pregnancy until 6-weeks postnatally (perinatal transmission, which refers to all infections detected prior to 6 weeks postpartum), and optionally until complete weaning (prevalent and incident postnatal transmission). Briefly there were 3 main health states: No HIV infection, HIV-infection, and death. The HIV-infection state were then subdivided into two sub-states :chronic HIV infection (CD4 count ≥ 350 cells/mm3), acute HIV infection (CD4 count < 350 cells/mm3 who themselves were divided into sub-states according to breastfeeding, ART treatment and/or PMTCT intervention. The fundamental unit of time in the simulation was a month. Transition probabilities from one state to another were constant over time and based on observed data in Cameroon: the prevalence of HIV, access to antenatal care, coverage of maternal HIV testing, coverage of maternal CD4 cell count assessment and live birth rate. The MTCT HIV transmission probabilities depended on the timing of the mother’s infection (prior/during pregnancy or lactation), CD4 cell count at different stages, the ART regimen and duration and the breastfeeding practices, thus the duration spent in each health state (see Additional file 1 for detailed calculations).

### Baseline input data

Baseline inputs used to characterize our hypothetical cohort of pregnant and lactating women were derived from different data sources: current national surveys from National AIDS Control Committee and Cameroon’s National Institute of Statistics [18, 19], clinical trials and cohort studies conducted in Cameroon or other resource-limited settings in the absence of national data [20–23]. All data used are summarised in Table 1. We also assumed that HIV infection is associated with a lower fertility among HIV-infected women [24, 25]. The proportion of live births issued from an HIV-infected woman was estimated taking this fact into account, as well as the fact that woman may die during pregnancy.

**Table 1:** Pregnant women population groups and model key parameters according to the observed data in the different age groups

Age groups (years)	15–49	15–19	20–24	25–29	30–34	35–39	40–44	45–49	Data source
Total number of pregnant women expected	995, 533	231, 982	202, 093	173, 223	125, 437	108, 513	80, 638	73, 647	Cameroon [18, 19]
HIV prevalence, %	5.6	2.0	3.4	7.6	7.3	10.0	7.1	6.4	Cameroon [18]
Access to Antenatal Care), %	36.5								Cameroon [19]
Prenatal HIV testing, %	29.3								Cameroon [19]
Receipt of maternal HIV test result, %	92.4								Cameroon [19]
Mortality during pregnancy, %	4								Cameroon [19]
Live birth among HIV uninfected women, %	95								Cameroon [9, 19]
Live birth among HIV infected women, %		94.6	72.7	68.9	63.3	58.4	52.0	51.9	[17, 26]
ARV prophylactic coverage for HIV infected pregnant women, %	20.3								Cameroon [19]
CD4 cell count quantification for HIV infected women, %	6.9								Cameroon [19]
Pregnant women ART eligible in ANC, %	60								Cameroon [19]
ART coverage for eligible women in ANC, %	25								Cameroon [19]
HIV MTCT rate at 6-week in the absence of MTCT, %	22								Sub-saharan Africa [23, 28]
Treatment Effectiveness Expected									[21, 23]
-Short course ARV (% reduction)	86.7								
-Long course ARV/ ART (% reduction)	93.7								
Rate of breastfeeding exposure among HIV exposed children known at birth (%)	46								Cameroon, Pediacam^a^ 1, CIRCB field studies^b^
Rate of breastfeeding exposure among children not known to be HIV exposed at birth, (%)	97								Cameron [18]
Intra-partum incidence of HIV infection (per 100 woman-year of follow-up), %		2	2	1.6	1.3	1	0.6	0.6	Sub-saharan Africa [39–41]
Incident HIV infections among lactating women, %	2.0								Zimbabwe [27, 31]
Proportion of children still exposed to breastfeeding (age in months), %									Cameroon [18]
[0,2[	97.5								
[2,4[	98.9								
[4,6[	96.0								
[6,9[	95.9								
[9,12[	90.5								
[12,18[	70.2								
[18, 24]	29.5								
Rate of exclusive breastfeeding among non HIV exposed children (age in months), %									Cameroon [18]
[0,2[	31.2								
[2,4[	23.0								
[4,6[	10.6								
[6,9[	1.9								
[9,12[	0.8								
[12,18[	0.7								
[18, 24]	0.0								
Rate of exclusive breastfeeding among HIV exposed children (age in months), %									Cameroon,
									Pediacam^a^ 1, CIRCB field studies^b^
[0,2[	86.6								
[2,6[	75.9								
[6,12[	50.4								
[12, 18]	0.0								
Proportion of HIV exposed children who received postnatal ART, %	9.2								Cameroon [19]
Rate of mother’s retention in ART treatment after delivery									Sub-saharan Africa [42]
[0,6[	79.1								
[6,12[	75.0								
[12, 24]	61.6								

*ANC* antenatal care, *ART* antiretroviral therapy, *ARV* antiretroviral, *MTCT* mother-to-child transmission; ^a^Pediacam is a multisite cohort study started in Cameroon in November 2007 with two main objectives: to study the feasibility and effectiveness, of early antiretroviral multi-therapy offered systematically to HIV-infected infants before 7 months of age; and to evaluate the humoral response of these children to vaccines of the Expanded Program of Immunization; ^b^Unpublished Early Infant Diagnosis of HIV data located at CIRCB

### Model structure: calculation of mother-to-child transmission probabilities

Mother-to-child-transmission (MTCT) of HIV can mainly occur during the second and third trimester of pregnancy, during delivery or breastfeeding [1]. Indeed, HIV transmission through breastfeeding has emerged as a substantial mode of MTCT among African breastfeeding populations and can occur in two different circumstances: among HIV prevalent mothers HIV-infected at delivery and among incident mothers HIV-infected while lactating. The risk of transmission through breastfeeding is cumulative according to the duration of breastfeeding and the longer the duration of breastfeeding, the greater the transmission risks [26–29]. Thus, we estimated three MTCT probabilities, using data from MTCT studies among pregnant and breastfeeding populations in Africa: 1/ the perinatal transmission probability at 6-week, 2/ the postnatal transmission probability and 3/ the postnatal transmission probability in those born to incident HIV-mothers who seroconverted while lactating. Additionally, we hypothesized that each of these three MTCT probabilities varied according to the maternal age group and CD4 count. Infants HIV status was computed using these estimated MTCT probabilities.

Details regarding the calculation of live births rate among HIV infected woman and the calculation of MTCT probabilities are available in an additional text file (see Additional file 1, which describes models used for the calculation of MTCT probabilities and live birth rate among HIV infected women).

### Rates of mother-to-child transmission of HIV and population size of new paediatric HIV infections

**Fig. 1:**
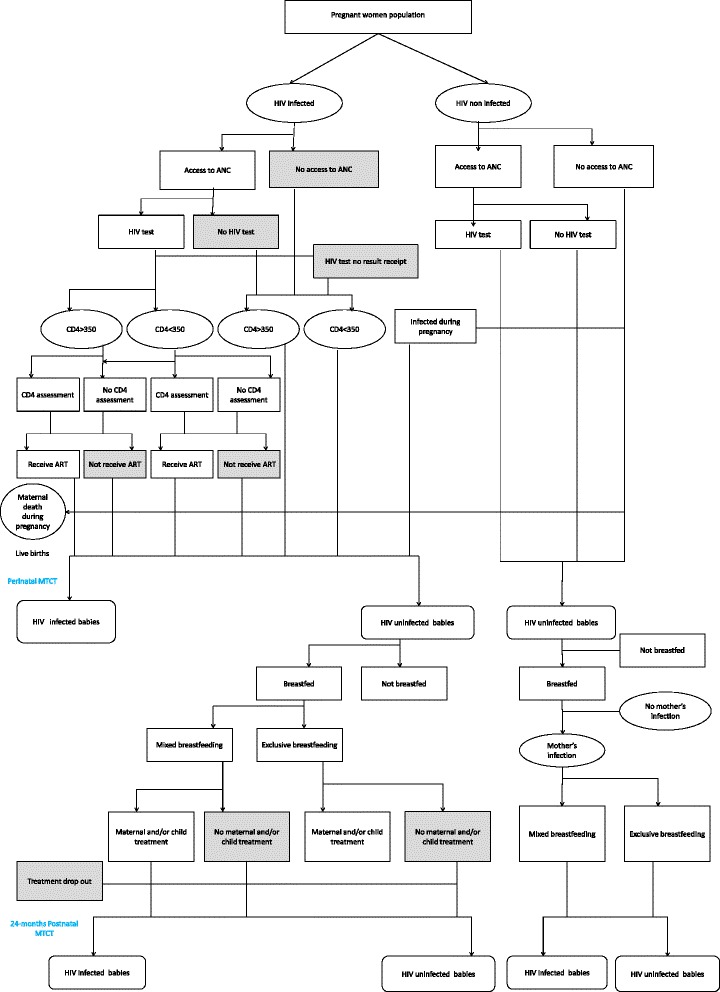
PMTCT cascade. This figure shows the different state between pregnancy and delivery, then delivery and breastfeeding cessation, including PMTCT services offering: gray colour highlights the missed opportunities in the PMTCT process. Each oval represents a maternal health state, rounded rectangle represents child health state and rectangle represents clinical events and breastfeeding practices

Using our model we simulated 1, 000 cohorts of the population size of pregnant women expected in Cameroon in 2011, through each state of the PMTCT cascade described in Fig. 1.

We calculated, using a Monte Carlo approach simulation, the MTCT rates including perinatal and postnatal transmission rates among prevalent HIV-infected mothers at birth and the postnatal transmission rate among incident mothers for these 1000 cohorts. We first modelled the probability for a mother to infect her infant, used that probability to estimate a number of HIV-infected infants born to mothers in the simulated cohort and divided that number by the number of children at risk of being HIV-infected in that cohort. Specifically, the final MTCT risks were calculated as follows: ***Perinatal transmission rate*** is equal to the average number of HIV-infected children at 6-weeks born alive to prevalent HIV-infected mothers divided by the total average number of live births from prevalent HIV-infected mothers. ***Postnatal transmission rate*** is equal to the average number of children HIV-uninfected at 6-weeks who become infected beyond through breastfeeding, divided by the total average number of children born alive to prevalent and incident HIV-infected mothers. Postnatal *t* ***ransmission rate due to incident infection among lactating mothers***: this rate is equal to the average number of breastfed HIV-infected children born alive to mothers HIV-uninfected at delivery but who seroconverted during breastfeeding, divided by the total average number of breastfed children born alive to incident HIV-infected mothers. ***Population size of new paediatric HIV infections*** is equal to the total average number of live born children from an HIV-infected prevalent mother at birth who become HIV-infected perinatally or during the breastfeeding period and the number of children HIV-infected through breastmilk from an incident HIV-infected lactating mother.

Finally, we examined the impact of different levels of uptake of PMTCT services on the MTCT rates and the number of new paediatric infections. Three scenarios were considered. First, we considered 100 % uptake and with a 100 % retention on treatment during the whole breastfeeding period. This meant that all pregnant women had access to antenatal care (ANC), HIV testing and counselling, disclosure of their HIV results; those who were infected had a CD4 assessment and were initiated on ART prophylaxis during pregnancy until the end of breastfeeding, as well for their child. In the second scenario, we considered a 90 % uptake of PMTCT services, described as 100 % access to ANC, 90 % HIV testing and counselling, a 100 % of HIV testing result disclosure and a 90 % rate of ART prophylaxis during pregnancy. The third scenario meant 80 % uptake of PMTCT services, described as 100 % access to ANC, 80 % HIV testing and counselling, a 100 % of HIV testing result disclosure and an 80 % rate of ART prophylaxis during pregnancy.

95 % confidence intervals of perinatal, postnatal and postnatal due to maternal incident infection rates, as well as the population size of new paediatric infections were derived from the 0.025 and 0.975 quintiles of 1000 subpopulation generated by bootstraps.

### Sensitivity analyses

Sensitivity analyses of the results of the simulation model were conducted by varying in suitable intervals some key parameters in the prevention of MTCT of HIV, as described in Table 2. Sensitivity analyses were performed by varying these parameters in specific intervals based on the lowest and highest published values. For each key parameter 1000 values were randomly drew within the corresponding range. For each of these 1000 sets of randomly drawn parameter we simulated one cohort of the population size of pregnant women expected in Cameroon in 2011 and the associated MTCT rates were calculated.

**Table 2:** Range of Model parameters for sensitivity analyses (results expressed in %)

Key parameters	Base case value	Range for sensitivity analyses	Data sources
Access to ANC	36.5	36–85	[18, 19]
HIV testing in ANC	80.1	80–100	[19]
ARV prophylactic coverage in ANC for HIV-infected women	67	50–74	[2, 19]
CD4 cell count quantification for infected women in ANC	22.9	22–100	[19]
ART eligible in ANC	60	40–60	[19]
ART coverage for eligible women in ANC	25	25–32	[3, 19]
Natural history of HIV MTCT at birth	22	15–25	[23, 28]
Treatment Effectiveness			[21, 23]
-Short course ARV (reduction)	86.7	86–95	
-Long course ARV/ ART (reduction)	93.7	93–95	
Incidence rate among postpartum lactating women	2.0	0.2–3.8	[31]

*ANC* antenatal care, *ART* antiretroviral therapy, *ARV* antiretroviral treatment, *MTCT* mother-to-child transmission

In this work, all results were performed using R statistical software (*R Core Team (2013). R: A language and environment for statistical computing. R Foundation for Statistical Computing, Vienna, Austria. URL* http://www.R-project.org/.).

## Results

The simulation model was performed for the population of 995, 533 pregnant women expected in Cameroon in 2011 [19].

### Base-case analyses: fixed values of PMTCT cascade

Available data showed a low rate of PMTCT services utilization (i.e. uptake of all components of the PMTCT cascade) everywhere in Cameroon, ranging from 6.9 % to 36.5 % in 2011 [19].

Table 3 presents the results of simulated mother-to-child transmission rates of HIV in both prevalent and incident HIV-infected mothers according to the observed coverage of PMTCT services in Cameroon in 2011. The overall MTCT rate, in both prevalent and incident HIV-infected mothers, was estimated to be 22.1 % (95 % Confidence Interval (CI): 18.6 %–25.2 %) in 2011, resulting in 10, 403 new paediatric HIV infections (95 % CI: 9, 054–13, 345).

**Table 3:** Simulated MTCT rates of HIV in prevalent and incident maternal population according to the observed coverage of PMTCT services in Cameroon in 2011

MTCT circumstances	Estimated number of incident paediatric infections [95 % Confidence Interval]	Estimated transmission rate (%)
Prevalent HIV-infected pregnant women		
Perinatal rate (<6 weeks of age)	3, 758 [3, 033–5, 077]	12.1 [8.8–15.1]
Postnatal rate (>6 weeks of age to 24 months)	3, 484 [2, 687–5, 286]	13.3 [9.3–17.8]
Postnatal rate among incident maternal infection during breastfeeding	3, 161 [2, 337–4, 679]	20.8 [14.1–26.9]
Total	10, 403 [9, 054–13, 345]	22.1 [18.6–25.2]

*MTCT* mother-to-child transmission, *PMTCT* prevention of mother-to-child transmission

Estimates of the perinatal MTCT rate was 12.1 % (95 % CI: 8.1 %–15.1 %), leading to 3, 758 new paediatric infections at 6-week of life (95 % CI: 3, 033–5, 077). During the postnatal period, the MTCT rate issued from prevalent HIV-infected women at delivery was 13.3 % (95 % CI, 9.3 %–17.8 %) corresponding to 3, 484 (95 % CI: 2, 687–5, 286) new paediatric infections. Among mothers who seroconverted during the breastfeeding period, the postnatal MTCT reached 20.8 % (95 % CI: 14.1 %–26.9 %), corresponding to 3, 161 (95 % CI: 2, 337–4, 679) new paediatric infections.

### Different PMTCT utilization coverage

Table 4 presents the results of three simulated scenarios of MTCT rates of HIV and number of new infections in prevalent maternal population according to the observed coverage of PMTCT services in Cameroon in 2011in case of 100 %, median 90 % and more likely of 80 % uptake of PMTCT services.

**Table 4:** Simulated scenarios of MTCT rates of HIV in prevalent maternal population according to the fixed coverage of PMTCT services in Cameroon in 2011

100 % of PMTCT services coverage		
MTCT circumstances	Estimated number of incident paediatric infections [95 % Confidence Interval]	Estimated transmission rate (%)
Perinatal rate (<6 weeks of age)	165 [99–398]	0.4 [0.2–1.1]
Postnatal rate (>6 weeks of age to 24 months)	149 [99–298]	0.8 [0.5–1.6]
Total	316 [198–631]	0.9 [0.5–1.7]
90 % of PMTCT services coverage		
Perinatal rate (<6 weeks of age)	509 [199–995]	1.4 [0.5–2.8]
Postnatal rate (>6 weeks of age to 24 months)	205 [99–497]	1.3 [0.5–3.0]
Total	711 [306–1, 187]	2.0 [0.9–3.2]
80 % of PMTCT services coverage		
Perinatal rate (<6 weeks of age)	1, 150 [497–1, 891]	3.3 [1.3–5.6]
Postnatal rate (>6 weeks of age to 24 months)	342 [99–696]	2.1 [0.6–4.4]
Total	1, 505 [895–2, 354]	4.3 [2.4–6.7]

*MTCT* mother-to-child transmission *PMTCT* prevention of mother-to-child transmission

When the model was run for a 100 % level of consumption of PTMCT services among prevalent HIV pregnant women, we estimated the MTCT rate of HIV at 6-weeks to be 0.4 % (95 % CI: 0.2 %–1.1 %), corresponding to 165 (95 % CI: 99–398) new paediatric perinatal infections. The postnatal transmission rate would be 0.8 % (95 % CI: 0.5 %–1.6 %), corresponding to 149 (95 % CI: 99–298) new paediatric postnatal infections. Thus, the global MTCT rate issued from prevalent HIV infected mothers would be 0.9 % (95 % CI: 0.5 %–1.7 %), with a total number of new paediatric infections of 316 (95 % CI: 198–631).

Assuming that PMTCT services were used at 90 %, the MTCT rate of HIV at 6-weeks would be 1.4 % (95 % CI: 0.5 %–2.8 %), corresponding to 509 (95 % CI: 199–995) new paediatric perinatal infections, and the postnatal transmission rate would be 1.3 % (95 % CI: 0.5 %–3.0 %), corresponding to 205 (95 % CI: 99–497) new paediatric postnatal infections. In this scenario, the global MTCT rate issued from prevalent HIV-infected mothers would be 2.0 % (95 % CI: 0.9 %–3.2 % with a total of new paediatric infections reaching 711 (95 % CI: 306–1, 187).

When running the model for a 80 % uptake of PTMCT services, the MTCT rate at 6-weeks would be 3.3 % (95 % CI: 1.3–5.6), corresponding to 1, 150 (95 % CI: 497–1, 891) new paediatric perinatal infections, and the postnatal transmission would be 2.1 % (95 % CI: 0.6 %–4.4 %) corresponding to 342 (95 % CI: 99–696) new paediatric postnatal infections. In this scenario, the global MTCT rate issued from prevalent HIV infected mothers would 4.3 % (95 % CI: 2.4 %–6.7 %), with a total number of new paediatric infections to be 1, 505 (95 % CI: 895–2, 354).

Hence, for prevalent HIV-infected mothers, reaching 100 % uptake of PMTCT services would lead to a 96.9 % (95 % CI: 93.9 %–98.0 %) reduction of new paediatric infections in 2011. When PMTCT services are used at 90 %, the new paediatric infections rate would be reduce by 93.1 % (95 % CI: 88.5 %–97.0 %) and by 85.5 % (95 % CI: 77.3 %–91.3 %) if PMTCT services are used at 80 %.

### Sensitivity analyses

When varying identified key parameters in the model, the mean perinatal transmission rate was 10.6 % (95 % CI: 6.2 %–15.5 %), the mean postnatal transmission rate among prevalent mothers was 12.3 % (95 % CI: 8.1 %–16.5 %) and the mean postnatal transmission rate among incident HIV-infected lactating mothers was 20.1 % (95 % CI, [11.3–29.5]). The population size of new paediatric infections in 2011 was 9, 131 cases (95 % CI: 4, 279–14, 834). Simulating improvements in coverage of prenatal services, prenatal HIV testing, CD4 cell count assessment among HIV-infected ARV for eligible women, PMTCT prophylactic services, improvement of treatment effectiveness, and the reduction of the incidence of HIV among lactating women led to the reduction of perinatal and postnatal transmission rates.

## Discussion

We developed a discrete event simulation model with a stochastic structure to estimate the rates of MTCT of HIV and the number of new paediatric HIV infections in 2011 in Cameroon cumulating the two situations of prevalent HIV-infected mothers at delivery and incident HIV-infected mothers while lactating. First, despite the PMTCT services implementation, we still observed in 2011 an overall residual global MTCT rate that reached 22 %. Second, in this context of PMCT interventions, our results highlight that most of paediatric HIV infections occurred postnatally, through breastfeeding, and this was particularly high among the mothers who acquired HIV during lactation. Finally, in scenarios where the uptake of PMTCT is high i.e.100 %, 90 % and 80 %, we could reach significant reductions of MTCT by 96.6 %, 93.1 % and 85.5 % respectively.

The results of this simulation model report an overall MTCT risk of 22 % in 2011 which remains high and consistent with the 24 % reported by the UNAIDS’ Global Plan that same year [7, 30]. We explain this high rate by a limited access to PMTCT interventions coupled with a low uptake of these services at different steps of the PMTCT cascade. Despite efforts towards eliminating new paediatric infections, Cameroon still faces limited availability of PMTCT services in primary health-care centres. In 2011, the country opted for the WHO Option A regimen for PMTCT prophylaxis. However, only 20 % of identified HIV-infected pregnant women received a PMTCT prophylaxis [19], and 27 % of their infants received a postnatal ART for prophylaxis [30]. It is urgent to expand the availability of PMTCT services within ANC and to improve the retention and access to services delivery over the whole MTCT period at risk, i.e. until the complete cessation of breastfeeding.

In our study, we also found that the risk of MTCT was highest in women who acquired HIV infection in the post-partum period. This result has already been reported in other settings [27]. Indeed, a study conducted in Zimbabwe showed that MTCT was 4.6 times higher in mothers who had seroconverted during breastfeeding compared to those who had been tested HIV-positive prior to delivery [27]. We explain this by a peak of maternal breast-milk viral load after the maternal HIV primo-infection [27, 31]. In our model, maternal infection during breastfeeding contributed significantly, for one-third, to the paediatric HIV burden. This result has been reported by other modelling studies, including one in South Africa where the proportion of MTCT was 26 % in mothers who seroconverted after their first ANC visit [32, 33]. Another survey conducted in Mozambique, reported an MTCT rate in mothers who seroconverted during breastfeeding of 21 % [34]. Nevertheless, despite a high HIV post-partum incidence in high prevalence settings in Africa, the HIV testing coverage in women in late pregnancy or in the postpartum period remains uncommon as testing practices often focus on the first ANC. In high HIV prevalence settings, early identification of incident infection in postpartum women and immediate testing of their exposed children is critical for initiating ART prophylaxis to prevent infection in infants and initiating early ART for those HIV-infected infants. Repeated HIV testing of HIV-uninfected mothers around delivery could allow identifying newly infected women and their exposed infants [35]. However, in resource-limited settings, the turnover period of these tests is often counted in weeks and the mother has already infected her child. In this context, it is primarily essential to focus on developing innovative primary HIV prevention programmes targeted on breastfeeding women and their partners [36]*.* Strategies to control postnatal MTCT should include community, behavioural and biomedical interventions focused to the vulnerable population of breastfeeding women.

According to the different PMTCT utilization rate scenarios analysed, we also demonstrated that the Global Plan target of reducing new paediatric infections by 90 % could be achieved if all pregnant women used PMTCT services at each step of the PMTCT cascade. This result is consistent with another modelling study conducted using the Spectrum model, that reported a reduction of 79 % in new paediatric infections in 25 countries, including Cameroon [37]. Decreasing markedly HIV MTCT transmission rates and eliminating new infections requires achieving family planning, primary HIV prevention of childbearing age women, and universal coverage of effective prenatal and postnatal antiretroviral regimen. In practice, Cameroon has achieved considerable progress, and in 2012, the ART coverage for PMTCT was estimated to be 64 % [11]. Furthermore, Cameroon started implementing Option B+ at the national level in 2014. Indeed, most of the efforts on PMTCT have been focused on improving access to prenatal antiretroviral prophylaxis. However, antiretroviral prophylaxis alone is insufficient and it is essential to improve the primary prevention and access to family planning. According to the Cameroon demographic health survey, 24 % of women still have unmet needs for family planning [18]. Modelling studies have suggested that there will be significant reductions in the number of HIV-exposed infants if unmet needs are reduced [37]. Thus, it is essential to strengthen programmes that offer family planning services to both HIV-infected and uninfected pregnant women.

Methods for producing estimates have their limitations [38]. First, computer simulations simplify complex biological and operational processes and many assumptions involved some uncertainty. However, our results are consistent with those previously reported by other modelling studies, including the Spectrum model. Second, we used multiple-source data from different settings that were not all issued from Cameroon. This may have also impacted our assumptions in the estimation of transition probabilities between states. However, sensitivity analyses conducted found that the impact on estimates was minimal. In the other hand, our model don’t take into account of maternal death during up to two months postpartum period. Consequently, the number of new paediatric HIV infections due to postnatal transmission could be overestimated in our model. Finally, in this analysis, we did not consider different PMTCT antiretroviral regimens. Indeed, it is still largely unclear which option is the most effective when implemented in a public healthcare system, but we felt that our average estimate of efficiency was likely [37]. The originality of this work is that most of the input PMTCT services and coverage components were issued from the Cameroon, which provides here a useful estimation of the paediatric infections projections for policymakers at the national level. The data presented here represent a base case scenario against which we will compare more recent estimates as they become available, reflecting other interventions as they are implemented, including option B+ regimen for PMTCT prophylaxis.

## Conclusion

Despite access to PMTCT services, paediatric HIV infections remain a concerning problem in Cameroon in 2011, with a high residual MTCT rate attributable to postnatal transmission. The national strategies planned to increase the PMTCT services coverage among HIV-exposed children, pregnant and lactating HIV-infected women to at least 90 % for the 2011–2015 periods to substantially reduce new infant HIV infections. However, to reach the goal of the ‘virtual elimination’ of paediatric HIV, the national strategic plan needs to first fix ANC access and delivery of antenatal HIV testing to 100 % for prevalent HIV-infected mother before delivery. An additional effort should be made to encourage pregnant women to use PMTCT services at all levels, at least until a complete breastfeeding cessation. Then, a substantial effort needs to be done to prevent HIV incidence in lactating women. This recurrent modelling informed by the national statistics will be helpful in guiding the national policy to PMTCT in Cameroon.

## Additional file

Additional file 1:
**Methods of calculation of mother-to-child transmission probabilities and live births rate among HIV positive women.** This additional file is a .docx file which contains description of mathematical models used to estimate mother-to-child transmission probabilities. It is also described here how live births rate among HIV positive women were calculated. (DOCX 37 kb)

## Abbreviations

ANC: Antenatal Care
ART: Antiretroviral Therapy
CIRCB: Centre International de Référence Chantal Biya pour la recherche sur la prévention et la prise en charge du VIH/SIDA
MTCT: Mother To Child Transmission
PMTCT: Prevention of Mother To Child Transmission
