# Supplementary material for: Estimating mother-to-child HIV transmission rates in Cameroon in 2011: a computer simulation approach
Source: BMC Infect Dis. 2016 Jan 12;16:11. doi: 10.1186/s12879-016-1336-2 (PMC4709976; doi:10.1186/s12879-016-1336-2)
Supplement: Additional file 1: — Methods of calculation of mother-to-child transmission probabilities and live births rate among HIV positive women. This additional file is a .docx file which contains description of mathematical models used to estimate mother-to-child transmission probabilities. It is also described here how live births rate among HIV positive women were calculated. (DOCX 37 kb) [file 12879_2016_1336_MOESM1_ESM.docx]

**Estimating Mother-to-child HIV transmission rates in Cameroon in 2011: a computer simulation approach**

Hermine L. Nguena Nguefack^1§^, Henri Gwet^1^, Sophie Desmonde^2,3^, Odile Ouwe Missi Oukem-Boyer^4, 5^, Céline Nkenfou^4^, Mathurin Téjiokem^6^, Patrice Tchendjou^6^, Irénée Domkam^4^, Valériane Leroy^2,3^, Ahmadou Alioum^2,3^ for the Inserm U897 Modeling Infectious Diseases in Low-Income Countries Study Group

1. National Advanced School of Engineering, The University of Yaoundé I, Yaoundé, Cameroon
2. Inserm, U897, Bordeaux, France
3. Bordeaux School of Public Health, The University of Bordeaux, Bordeaux, France
4. Centre International de Référence Chantal Biya (CIRCB) pour la recherche sur la prévention et la prise en charge du VIH/SIDA, Yaoundé, Cameroun
5. Centre de Recherche Médicale et Sanitaire (CERMES), member of Réseau International des Instituts Pasteur, Niamey, Niger
6. Centre Pasteur du Cameroun, member of Réseau International des Instituts Pasteur, Yaoundé, Cameroun

**Methods of calculation of mother-to-child transmission probabilities and live births rate among HIV positive women**

**Model structure: calculation of mother-to-child transmission probabilities**

MTCT of HIV can mainly occur during the second and third trimester of pregnancy, during delivery or breastfeeding [1]. The risk of transmission through breastfeeding is cumulative according to the duration of breastfeeding. Transmission can take place at any time during breastfeeding, and the longer the duration of breastfeeding, the greater the transmission risk [2, 3]. Risks of MTCT during pregnancy and breastfeeding were calculated from MTCT studies among breastfeeding populations in Africa. In the model, each baby born from a prevalent HIV-positive woman at birth has an associated risk of being infected, which may vary from one risk group to another. Here the risk groups were defined according to mother’s age.

***Perinatal transmission***

In our model, we assumed that if the mother does not receive any antiretroviral prophylaxis then the perinatal transmission probability depends on maternal CD4 count (<350 cells/mm^3^ or >350/mm^3^ cells) during pregnancy and whether or not the mother became infected during the current pregnancy. If the mother received prophylaxis, the perinatal transmission depends on maternal CD4 count (<350 cells or >350 cells) and type of prophylaxis that she received [4]. Additionally, we assumed that recent infection of the mother during pregnancy and maternal CD4 count<350 can double the risk of HIV transmission from mother to her baby [2, 4].

Let p_i_ be the natural history of mother-to-child HIV transmission for a risk group *i* and *Z_r_* (r=1, 2) the effectiveness of a treatment intervention r (prophylactic treatment or ART) in terms of reducing the MTCT rate of HIV. L_i_ indicates a maternal CD4 count less than 350 and T_i_ the occurrence of maternal HIV infection during current pregnancy;

The probability Q^1^_il_ that a child born alive from an HIV-infected woman who received a treatment *r* or not during pregnancy and/or delivery in a risk group *i* is truly infected at birth is given by:

$$Q_{i}^{1}=\left\{ \begin{matrix} \begin{matrix} p_{i}\left( 1+T_{i}+L_{i}-T_{i}L_{i} \right) & \end{matrix} & \text{if mother does not receive treatment} \\ p_{i}(1+L_{i})\left( 1-Z_{r} \right) & \text{ }if mother has treatment \end{matrix} \right. (1)$$

***Postnatal transmission***

HIV transmission through breastfeeding has emerged as substantial mode of MTCT among African breastfeeding populations and could occur in two different circumstances, among HIV prevalent mothers at delivery and among incident lactating mothers [5–9]; the longer the duration, the greater the cumulative risk of postnatal HIV transmission. Advanced HIV maternal immunodeficiency is also a major risk factor [5, 10]. Furthermore, exclusive breastfeeding has been reported to reduce postnatal HIV transmission risk by 50% compared to mixed breastfeeding [11]. Therefore, postnatal MTCT rate is assumed to depend on pattern of breastfeeding (exclusive or mixed), maternal CD4 count, duration of breastfeeding and maternal ART coverage. Published data reported an overall constant over time risk of postnatal acquisition of HIV of 5.1 percent child-years in exclusively breastfed children (when the mothers was not exposed to ART) [5, 10, 12, 13] corresponding to a monthly risk of 0.425 percent child-months of breastfeeding. If breastfed infants are exposed to ART to prevent postnatal HIV transmission, the MTCT risk is reduced by approximately 72% [14]. Moreover, Rollins et al. showed that long-course maternal ART reduces the monthly risk of postnatal transmission of about 93% and short course of about 86% [15].

Consequently, let M_i_ be equal to 1 if baby received mixed breastfeeding and 0 otherwise; we noted by λ the monthly risk of transmission through breastfeeding, in the absence of any intervention;

The probability Q^2^_ilt_ that a not infected child at birth who is breastfed for *t* months becomes infected through breastfeeding is given by:

$Q_{\mathrm{ilt}}^{2}=\left\{ \begin{matrix} 1-\exp\left[ -\lambda\left( 1+M_{i}+L_{i} \right)t \right] & \text{if mother and child doest not received treatment} \\ 1-\exp\left[ -\lambda\left( 1+L_{i} \right)\prod_{r=1}^{l} \left( 1-Z_{r} \right)t \right] & \text{if mother and child received treatment and} t\leq t_{\text{treat}} \\ 1-\exp\left[ -\lambda\left( 1+L_{i} \right)\prod_{r=1}^{l} \left( 1-Z_{r} \right)t_{\text{treat}}-\lambda\left( 1+L_{i} \right)\left( t-t_{\text{treat}} \right) \right] & \text{if mother and child received treatment and} t>t_{\text{treat}} \end{matrix} \right.$ (2)

where t_treat_ is the duration of treatment for the mother and/or for child during breastfeeding; we considered the same duration of treatment for mother and child. We also assumed a maximal duration of breastfeeding of 24 months.

***Postnatal MTCT transmission due to HIV incident infection among mother while breastfeeding***

In high HIV prevalence context, HIV-uninfected at the time of delivery may become infected during the breastfeeding period [13]. In this case, an overall risk is estimated in the literature, rather than a monthly transmission risk. The first overall estimate of postnatal MTCT transmission among mothers HIV-infected while breastfeeding available is derived from a pooled analysis with an overall risk of 29% [7]*.*Consistently, it is reported that the breastfeeding associated transmission risk for mothers who seroconverted postnatally averaged 34.56 infant infections per 100 child-years during the first nine months after maternal infection, and declined to 9.50 during the next three months [13]. We assumed that this rate remains constant thereafter. Published data show that the pooled incidence rate of HIV infection during the postpartum period is 2.9 per 100 person-years (95% CI 1.8–4.0) and the stratified pooled incidence rate among lactating women is 2.0 per 100 person-years (95% CI 0.2–3.8) [16]. We assumed that the probability of MTCT in lactating mothers who seroconvert postnatally depend on the breastfeeding exposure, with a high breast milk HIV viral load. In fact, lactating women who are unaware of their HIV status may not be detected as HIV-infected before their next pregnancy.

Hence the probability Q^3^_it_ that a live-born child to a lactating woman HIV-seroconverted at time t_inf_ during breastfeeding, who has been breastfed for t months in a risk group *i* is truly infected during breastfeeding is given by:

$Q_{\mathrm{it}}^{3}=\left\{ \begin{matrix} \begin{matrix} 1-\exp\left[ \left( -\lambda_{1}(1+M_{i})\left( t-t_{\text{inf}} \right) \right) \right] & \end{matrix} & \mathrm{if}t \leq t_{\text{inf}}+9 \\ 1-\exp\left[ \left( -{9\lambda}_{1}\left( 1+M_{i} \right)-\lambda_{2}(1+M_{i})\left( t-t_{\text{inf}}-9 \right) \right) \right] & \text{if }t >t_{\text{inf}}+9 \end{matrix} \right.$ (3)

where λ_1_ is the monthly risk of transmission through breastfeeding during the firsts nine months after mother’s infection and λ_2_ the monthly risk of transmission between nine months and the end of breastfeeding. The final HIV status of the baby is governed by the probability Q^3^_it_ of transmitting HIV from an HIV-positive mother who seroconverted during breastfeeding to her baby.

**Calculation of live births rate among HIV positive women**

Based on formulas use in Spectrum [4], we first estimated the age-specific number of live births among HIV positive pregnant women for the year 2011.

Finally the live birth rate among HIV infected women by age was given by the ratio between the number of live births to HIV infected mothers on the total number of HIV infected pregnant women for each age group. For our calculations, the data used come from 2011 Demographic Health Survey (DHS) and National AIDS Control Committee (NACC) progress reports [17, 18].

**References**

[1] K. De Cock, M. Fowler, E. Mercier, I. De Vincenzi, J. Saba, E. Hoff, and et al, ‘Prevention of mother-to-child hiv transmission in resource-poor countries: Translating research into policy and practice’, *JAMA*, vol. 283, no. 9, pp. 1175–1182, 2000.

[2] World Health Organisation, *HIV transmission through breastfeeding : a review of available evidence : 2007 update*. 2008. ISBN 978 92 4 159659 6. Available:

http://whqlibdoc.who.int/publications/2008/9789241596596_eng.pdf?ua=1. Accessed 2013 december 18..

[3] V. Leroy, M. Newell, F. Dabis, C. Peckham, P. Van de Perre, and for the Ghent International Working Group on Mother-to-Child Transmission of HIV, ‘Late postnatal Mother-to-Child transmission of HIV-1 infection: author’s reply’, *Lancet*, vol. 352, p. 1630, 1998.

[4] J. Stover, ‘Spectrum: Methods for Estimating Mother-to-Child Transmission of HIV’. 06-May-2011.

[5] R. Becquet, R. Bland, V. Leroy, N. Rollins, D. Ekouevi, A. Coutsoudis, F. Dabis, H. Coovadia, R. Salamon, and M. Newell, ‘Duration, Pattern of Breastfeeding and Postnatal Transmission of HIV: Pooled Analysis of Individual Data from West and South African Cohorts’, *PLoS ONE*, vol. 4, no. 10, p. e7397, 2009.

[6] R. Nduati, G. John, D. Mbori-Ngacha, B. Richardson, J. Overbaugh, A. Mwatha, J. Ndinya-Achola, J. Bwayo, F. Onyango, J. Hughes, and Kreiss, ‘Effect of Breastfeeding an Formula Feeding On Transmission of HIV-1: A randomized Clinical Trial’, *JAMA*, vol. 283, no. 9, pp. 1167–1174, Mar. 2000.

[7] D. Dunn, M. Newell, A. Ades, and C. Peckham, ‘Risk of human immunodeficiency virus type 1 transmission through breastfeeding’, *Lancet*, vol. 340, pp. 585–588, Sep. 1992.

[8] A. Njom Nlend, C. Same Ekobo, B. Bagfegue Ekani, J. Epee Ngoue, S. Tetang Ndiang, and et al, ‘Preventing HIV-1 transmission in breastfed infants in low resource settings: early HIV infection and late postnatal transmission in a routine prevention of mother-to-child transmission program in Yaounde, Cameroon’, *J Trop Pediatr*, vol. 59, no. 5, pp. 387–392, 2013.

[9] V. Leroy, M. Newell, F. Dabis, C. Peckham, P. Van de Perre, and et al, ‘International multicentre pooled analysis of late postnatal mother-to-child transmission of HIV-1 infection. Ghent International Working Group on Mother-to-Child Transmission of HIV’, *Lancet*, vol. 352, no. 9128, pp. 597–600, 1998.

[10] The Breastfeeding and HIV International Transmission Study Group, ‘Late Postnatal Transmission of HIV-1in Breast-Fed Children: An Individual Patient Data Meta-Analysis’, *JID*, vol. 189, pp. 2154–2166, 2004.

[11] A. Coutsoudis, K. Pillay, E. Spooner, L. Khun, and H. Coovadia, ‘Infuence of infant-feeding patterns on early mother-to-child transmission of HIV-1 in Durban, South Africa : a prospective cohort study’, *Lancet*, vol. 354, no. 9177, pp. 471–476, Aug. 1999.

[12] P. Iliff, E. Piwwoz, N. Tavengwa, C. Zunguza, E. Marinda, K. Nathoo, L. Moulton, B. Ward, and J. Humphrey, ‘Early exclusive breasfeeding reduces the risk of postnatal HIV-1 transmission and increases HIV-free survival’, *AIDS*, vol. 19, pp. 699–708, 2005.

[13] J. Humphrey, E. Marinda, K. Mutasa, L. Moulton, P. Iliff, R. Ntozini, H. Chidawanyika, K. Nathoo, N. Tavengwa, A. Jenkins, E. Piwoz, P. Van de Perre, and B. Ward, ‘Mother to child transmission of HIV among Zimbabwean women who seroconverted postnatally: prospective cohort study’, *BMJ*, vol. 341, p. c6580, 2010.

[14] C. Kankasa, N. Nagot, N. Meda, J. Tumwine, A. Aku, D. Jackson, R. Vallo, T. Tylleska, and P. Van de Perre, ‘Infant Lopinavir/r Versus 3TC To Prevent Postnatal HIV-1 Transmission: The ANRS 12174 Trial’. Abstract 71, The 2014 Conference on Retroviruses and Opportunistic Infections (CROI).

[15] N. Rollins, M. Mahy, R. Becquet, L. Khun, T. Creek, and L. Mofenson, ‘Estimates of peripartum and postnatal mother-to-child transmission probabilities of HIV for use in Spectrum and other population-based models’, *Sex Transm Infect*, vol. 88, pp. i44–i51, 2012.

[16] A. Drake, A. Wagner, B. Richardson, and John-Stewart, ‘Incident HIV during Pregnancy and Postpartum and Risk of Mother-to-Child HIV Transmission: A Systematic Review and Meta-Analysis’, *PLoS Med*, vol. 11, no. 2, p. e1001608, 2014.

[17] Institut Nationale de la Statistique du Cameroun, ‘Enquête Démographique et de Santé et à Indicateurs Multiples (EDS-MICS) 2011’. Sep-2012. pp 71-88, 159-186, 219-223, 265-285. Available : <http://dhsprogram.com/pubs/pdf/FR260/FR260.pdf>.

[18] Comité National de Lutte Contre le SIDA du Cameroun, ‘Vers l’élimination virtuelle de la transmission du VIH de la mère à l’enfant à l’horizon 2015, rapport de progrès numéro 6, année 2011’, Rapport de progrès, 2012.
